# A symmetry mismatch unraveled: How phage HK97 scaffold flexibly accommodates a 12-fold pore at a 5-fold viral capsid vertex

**DOI:** 10.1126/sciadv.adg8868

**Published:** 2023-06-16

**Authors:** Alexis Huet, Bonnie Oh, Josh Maurer, Robert L. Duda, James F. Conway

**Affiliations:** ^1^ Department of Structural Biology, University of Pittsburgh School of Medicine, Pittsburgh, PA, USA.; ^2^ Department of Biological Sciences, Dietrich School of Arts and Sciences, University of Pittsburgh, Pittsburgh, PA, USA.

## Abstract

Tailed bacteriophages and herpesviruses use a transient scaffold to assemble icosahedral capsids with hexameric capsomers on the faces and pentameric capsomers at all but one vertex where a 12-fold portal is thought to nucleate the assembly. How does the scaffold orchestrate this step? We have determined the portal vertex structure of the bacteriophage HK97 procapsid, where the scaffold is a domain of the major capsid protein. The scaffold forms rigid helix-turn-strand structures on the interior surfaces of all capsomers and is further stabilized around the portal, forming trimeric coiled-coil towers, two per surrounding capsomer. These 10 towers bind identically to 10 of 12 portal subunits, adopting a pseudo-12-fold organization that explains how the symmetry mismatch is managed at this early step.

## Teaser

The assembly of a viral shell is a ballet tightly regulated by a versatile molecular scaffold.

## INTRODUCTION

Tailed double-stranded DNA bacteriophages and their eukaryotic counterparts, the herpesviruses, are the largest family of viruses on Earth, sharing capsid protein folds, icosahedral geometry, and assembly pathways (*1*). Capsids assemble first as a round precursor procapsid that changes to an angular mature capsid during DNA packaging. The icosahedral geometry is defined by pentameric capsomers of the major capsid protein (mcp) located at the vertices and a specific number of hexameric capsomers on the faces. However, symmetry is broken at a unique vertex occupied by an annular molecular pore called portal through which the viral DNA enters and leaves (*2*). Assembly of this large complex from hundreds of protein subunits requires transient association of an internal scaffolding protein or domain that acts similar to a chaperone and is absent from the mature capsid. Scaffolds appear to have multiple roles during procapsid assembly and may be an independent protein (as in phages λ, P22, T4, SPP1, and phi29 and the herpesviruses) or a scaffolding domain fused to the mcp N terminus (as in phages HK97 and T5). Without scaffolding proteins, the mcps of phages λ, P22, T4, and phi29 produce aberrant structures (*3*–*6*), while the HK97 mcp aggregates without its attached scaffolding domain (*7*). In herpes simplex virus (HSV) and phage P22, specific mutations in the scaffold block portal incorporation, suggesting a direct interaction between scaffold and portal (*8*, *9*). However, mechanisms underlying the scaffold’s functions remain unclear. Crystallography of the phage phi29 scaffold revealed α helices organized as a coiled coil (*10*), consistent with predictions based on the primary sequences of many phage internal scaffolds, including HK97 (*11*). Nuclear magnetic resonance analysis of the small mcp-binding domain of phage P22’s independent scaffolding protein revealed a charged helix-turn-helix motif consistent with Prohead assembly in vitro being dependent on ionic strength (*12*). Procapsid structures obtained by cryo–electron microscopy (cryo-EM) for phages P22, T7, SPP1, and 80α (*13*–*17*) do not reveal strong density attributable to the scaffold except for the mcp-binding regions beneath capsomers. This lack of scaffold density may be due to its temporary and flexible nature as well as its uncertain stoichiometry with the mcp. Phages such as HK97 with a scaffolding domain fused to the N terminus of the mcp have, de facto, a 1:1 ratio of scaffold:mcp, and earlier low-resolution procapsid reconstructions revealed projections from the capsid shell toward the interior of the capsid that were attributed to scaffold (*11*, *18*, *19*).

**Fig. 1.**
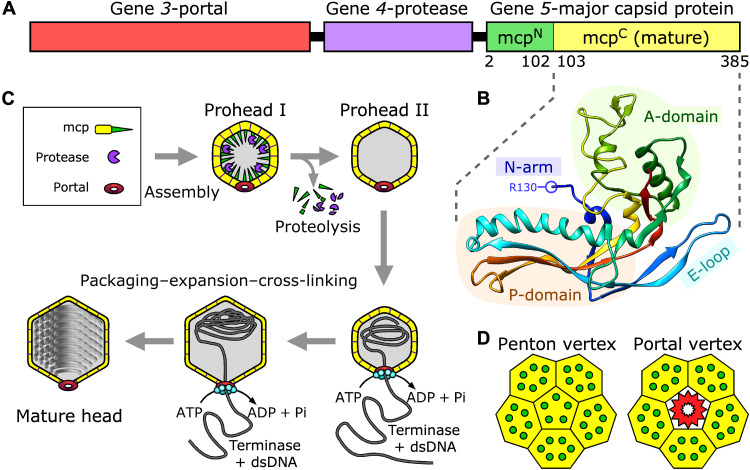
Phage HK97 capsid assembly. (**A**) Organization of the structural genes. A key feature is attachment of the scaffold domain to the N terminus of the mature mcp. (**B**) A diagram showing the structural elements of the HK97-fold as illustrated by the known structure of Prohead II [Protein Data Bank (PDB) ID: 3E8K], which lacks the scaffold domain. (**C**) The HK97 capsid assembly pathway is shown in cartoon form to illustrate the relationships between proteolysis, DNA packaging, and expansion. Cross-linking between mcps occurs late, during expansion of the shell. (**D**) Schematic comparing organization of the regular penton vertex to the portal vertex. ATP, adenosine triphosphate; ADP, adenosine diphosphate; dsDNA, double-stranded DNA; Pi, inorganic phosphate.

Phage HK97 is a highly tractable system to study capsid assembly. Expression of HK97 portal, protease, and mcp genes in its natural host leads to abundant production of assembled shells at sequential stages of assembly depending on which genes are included. These expression studies detailed the HK97 capsid assembly pathway while pioneering crystallographic structures of the portal-less expanded head (*20*), and Prohead II (*21*) revealed the eponymous HK97-fold in situ (Fig. 1, A and B), now known to be widespread and conserved. The mcp gene *5* encodes a 103-residue N-terminal scaffolding domain (mcp^N^) and a 281-residue C-terminal domain (mcp^C^) that exhibits the HK97-fold including a triangular A-domain; a P-domain, composed of a long spine α helix; an adjacent three-stranded β sheet; the flexible E-loop; and the N-arm. The N-arm is the N-terminal part of mcp^C^ to which the mcp^N^ scaffold domain attaches. Biophysical measurements indicate that mcp^N^ is largely α-helical (*22*), matching predictions that it is composed of two coiled-coil α helices followed by a third helix and a β strand. However, the mcp^N^ structure has not been determined.

Here, we focus on the uncleaved HK97 procapsid, Prohead I, that has intact scaffolding domains and a portal at one vertex (Fig. 1C) (*23*) to study the scaffold’s role at this critical step of capsid assembly. Using cryo-EM and asymmetric reconstruction methods similar to those used to visualize the portal vertices of herpesvirus (*24*), phi29 (*25*), and gene transfer agent (*26*), we determined the organization of scaffolding domains in situ and how they mediate interactions with the dodecameric portal complex. We explored whether the portal and surrounding hexons are in mutual register, and if so, whether the portal adopts local fivefold symmetry, as suggested by P22 portal structures (*27*), or whether the surrounding hexameric capsomers (hexons) deviate from their fivefold symmetry organization.

## RESULTS

### HK97 scaffold has multiple domains

**Fig. 2.**
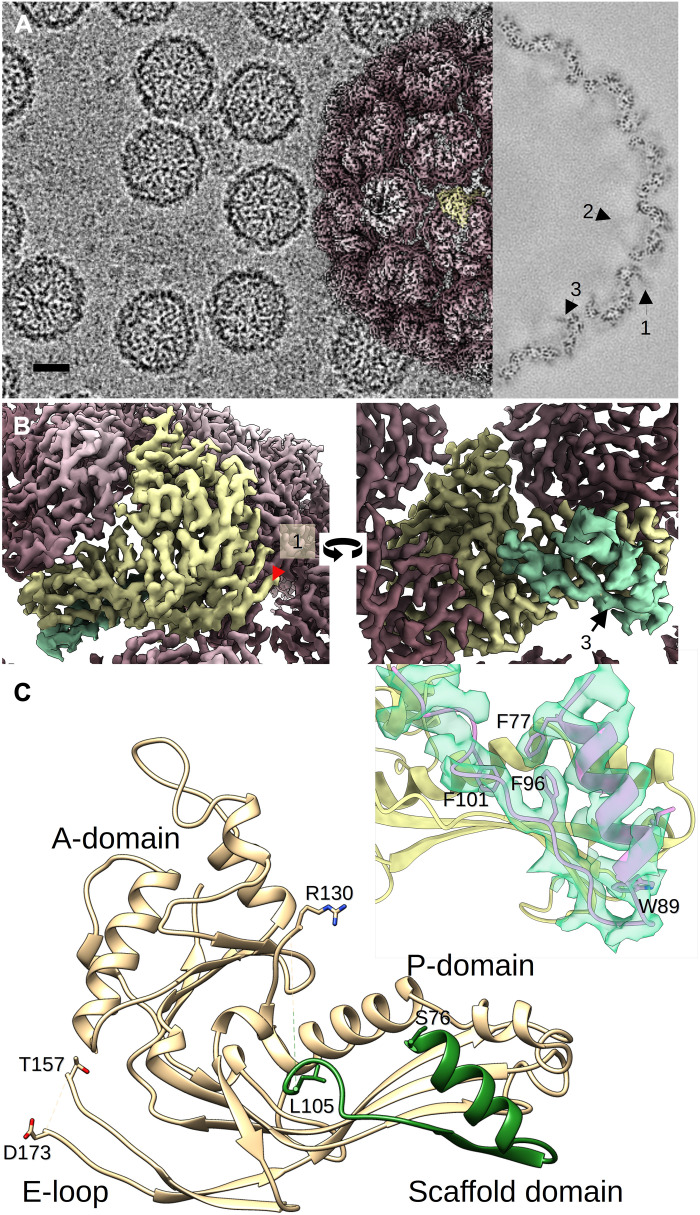
A portion of the scaffolding domain is visible under the capsomer of the HK97 Prohead I. (**A**) Portion of a representative cryo-micrograph of Prohead I. Scale bar, 25 nm. Inset (left): Surface rendition of the HK97 Prohead I density map at 3.5-Å resolution with icosahedral symmetry imposed. Inset (right): Central thin section through the density with three numbered densities marked by arrows that also are similarly marked in (B) and fig. S1. Arrow #1 points to the E-loop, and arrows #2 and #3 both point to parts of the scaffolding domain. Arrow #2 shows faint density corresponding to towers of scaffolding domain shown in fig. S1. (**B**) Views of the fivefold-symetric focused reconstruction of the penton vertex. Left: A view of the capsid exterior showing a hexon mcp^C^ subunit colored in yellow, with its scaffold domain beneath in light green. The resolved extent of the E-loop is indicated by a red arrowhead and arrow #1. Right: View of the same subunit from the capsid interior showing the density (in light green) that we assigned to the scaffolding domain (arrow #3). (**C**) Model of a hexon mcp subunit built using the penton-focused reconstruction (B), showing the mcp^C^ domain in light brown and the mcp^N^ domain in green. Inset: Visualization of the scaffolding domain model in its associated density to illustrate the fit of several large aromatic residue side chains.

We visualized the entire HK97 Prohead I by cryo-EM. An initial map with icosahedral symmetry imposed was solved to 3.6-Å resolution for exploring the global organization of the Prohead. Subsequent maps were derived using focused reconstruction directed at either the regular pentameric capsomer (penton) vertices or the portal vertex, with either 5-fold or 12-fold symmetry imposed or no symmetry at all. The icosahedrally averaged map reveals a well-defined HK97 procapsid shell shown in a surface view (Fig. 2A, left inset). A cross section of that map also shows strong density corresponding to the shell and reveals three particular densities marked by arrows (Fig. 2A, right inset). Arrow #1 denotes the visible part of the flexible E-loop that extends out from the capsid surface and is not completely resolved. The two other densities are beneath the capsomers and extend toward the capsid interior (arrows #2 and #3; Fig. 2A, right inset). These densities resemble those observed at lower resolution (*11*), and we ascribe them to the scaffold domain. To visualize the density marked by arrow #2, the map was low-pass–filtered and a view of the capsid interior (fig. S1) reveals one density beneath each penton with weak connections to the five penton subunits and one two-lobed density under each hexon, with each lobe connected to three hexon subunits (fig. S1, in dark green). These weak densities join to strong spoke-like densities (fig. S1, in light green, arrow #3, and Fig. 2B, right) that are absent from the interior surface of capsomers in the Prohead II structure (*21*). Our focused reconstruction of the penton vertices had sufficiently detailed side chain density to build a 30-residue model into the spokes (Fig. 2C, inset), which are composed of residues 76 to 103 of mcp^N^. This segment of scaffolding domain adopts a helix-turn-strand motif with residues S76 to S88 forming a rigid α helix lining the interior surface of the capsomer subunits, followed by a turn, and residues K92 to G97 adding an additional strand to the three-strand β sheet of the mcp^C^ P-domain. The final model (Fig. 2C) reveals that the density is discontinuous between residues L105 and R130, showing that the N-arm region is flexible. Hence, compared to Prohead II, the N-arm is not visible and the cleavage site, K103/S104, lies in a rather unstructured part of the shell.

**Fig. 3.**
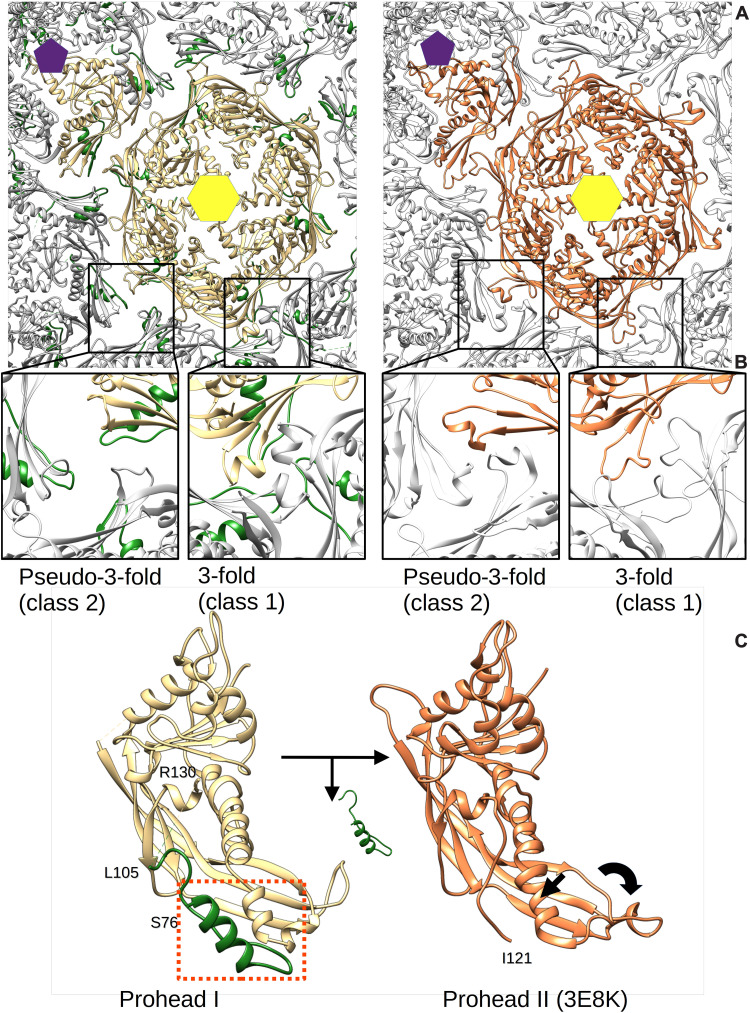
Removal of the scaffolding domain induces local and global conformational changes. (**A**) Views of the asymmetric units of Prohead I (left) and Prohead II (right) (PDB ID: 3E8K) in the context of their capsids. Hexon and penton centers are indicated by yellow and purple polygons, respectively. (**B**). Close-up views at a pseudo- and true threefold axis of Prohead I (left) and of Prohead II (right). The views have been slightly rotated compared to (A) to look toward the center of the capsid. (**C**) The conformation of the mcp subunit before (left) and after (right) removal of the scaffolding domain (green), showing a relaxation of the spine helix that may trigger displacement of the P-domain toward the pseudo-threefold axis (black arrows). A dashed orange box on the Prohead I model indicates the scaffold helix/spine helix interaction. Comparison of the left and right panels also shows that the N-arm in Prohead II (right) occupies approximately the same position as the scaffolding domain in Prohead I (left).

The asymmetric units of our Prohead I model and of Prohead II [Protein Data Bank (PDB) ID: 3E8K] are very similar in overall structure but have notable differences at the interior of the capsomers and at the intracapsomer local threefold interfaces (Fig. 3). In Prohead II after the removal of the scaffold, about 10 residues (~119 to 129) of the N-arms become visible in locations on hexons previously occupied by scaffold (Fig. 3C, compare left and right), reorganizing the local structure with probable consequences on the overall conformation. As defined previously (*28*), the HK97 shell has three structurally distinct threefold interfaces: the icosahedral threefold (class 1), a pseudo-threefold surrounded by hexons (class 2), and another pseudo-threefold surrounded by a penton and two hexons (class 3). In Prohead I, the ends of three P-domains do not interact with each other at the class 2 and 3 sites as they do in Prohead II (Fig. 3B and fig. S2). Note that unlike the crystallographic Prohead I model (*28*) where only the class 2 site exhibits a hole, our cryo-EM density of Prohead I showed holes at both the class 2 and 3 locations. The Prohead I crystals were grown from Proheads that had no portal but contained an inactivated protease and were resolved to only 5.2-Å resolution, so the origin of the structural differences is unclear. In our current model, the end of the P-domain adopts an arch-like structure perpendicular to the shell (fig. S2). This unique conformation appears to be supported by a neighboring intracapsomeric E-loop that folds against the P-domain (fig. S2). The arch motif of the P-domain also connects directly to the mcp^C^ spine helix that also forms a short coiled coil with the mcp^N^. As a consequence, removal of mcp^N^ might allow relaxation of the spine helix and drive the local threefold reorganization observed in Prohead II (*21*) (movie S1).

### The scaffolding domain mediates the symmetry mismatch at the portal vertex

**Fig. 4.**
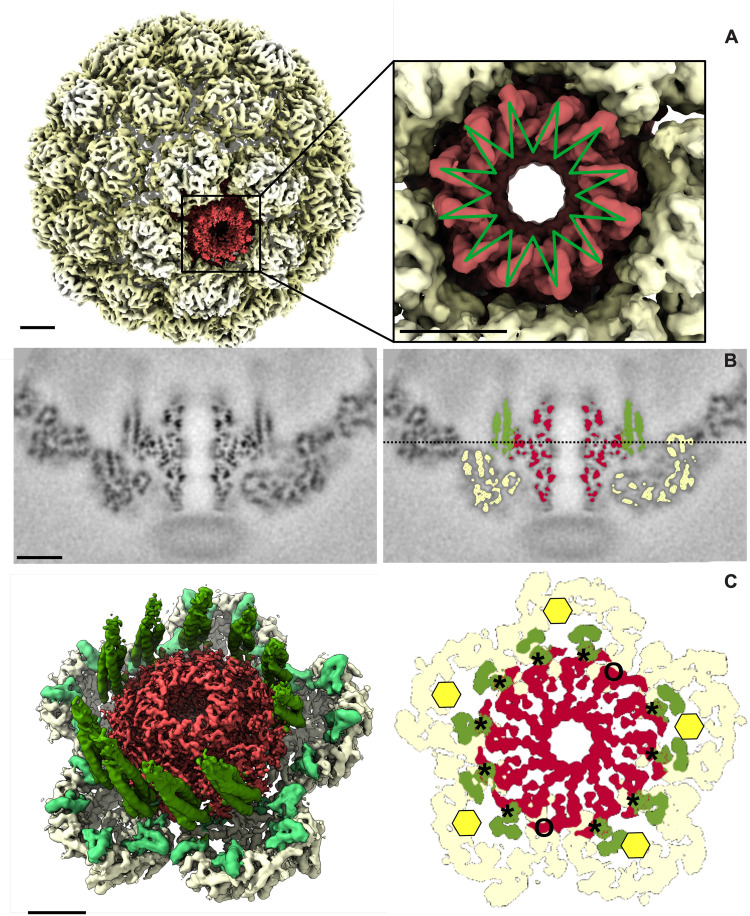
Asymmetric density map reveals organization of the portal vertex. (**A**) The clip domain of the portal (red) viewed from the capsid exterior (left) departs from strict 12-fold symmetry (green 12-fold star at the right). (**B**) Axial cross section through the portal vertex showing strong and detailed density for both the portal and shell in the absence of imposed symmetry. Furthermore, scaffolding domains are evident, as indicated by color at the right. Yellow, mcp^C^; green, mcp^N^; red, portal. (**C**) Left: Surface view from the capsid interior revealing 10 scaffolding domain towers consistent with coiled-coil trimers. Right: Section according to the dashed line in (B) showing that the 10 towers of scaffold (asterisks) interact at strictly equivalent positions around the portal, leaving two positions empty (circles). Scale bars, 5 nm.

**Fig. 5.**
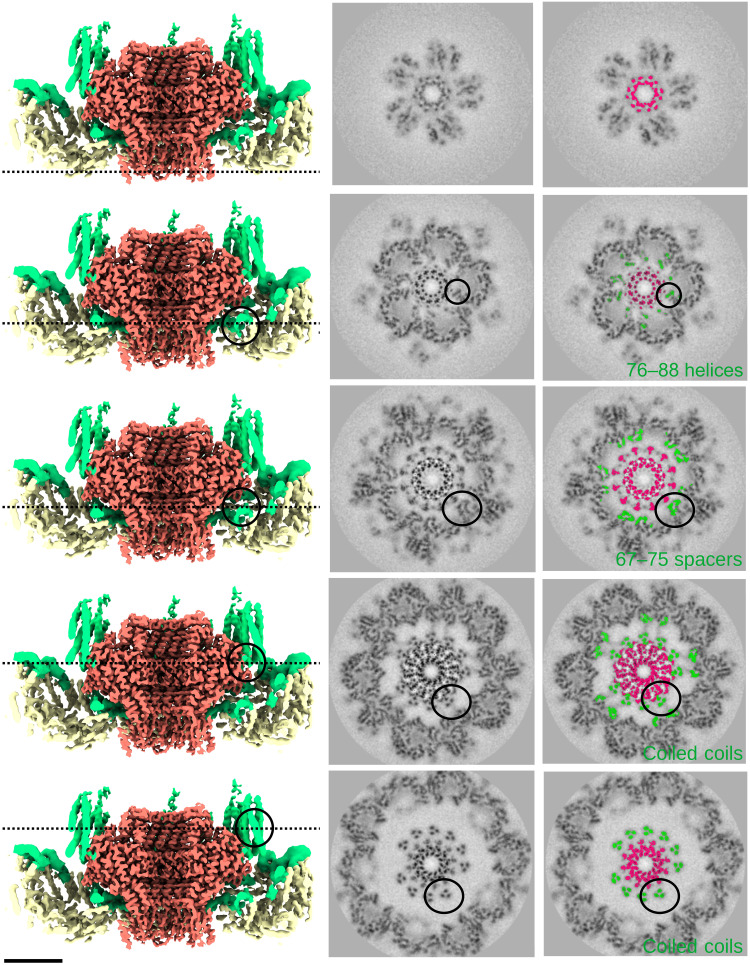
In Prohead I, the portal interacts mostly, if not only, with the scaffolding domain. The middle column shows grayscale sections through the asymmetric portal map that documents the contacts between the portal and surrounding structural elements. The left column shows surface views of the portal vertex after cutting away the front half through the central axis. The portal is colored red, mcp^N^ in green, and mcp^C^ in yellow. Black dashed lines through the images indicate the heights along the portal axis corresponding to the sections in the center column. The right column annotates the grayscale sections in the middle column by coloring the portal density in red and the scaffold density in green. The black circles highlight the scaffolding domain density visible in the cross section. The observed domain of the scaffold domain is colored in green (bottom right). Scale bar, 5 nm.

**Fig. 6.**
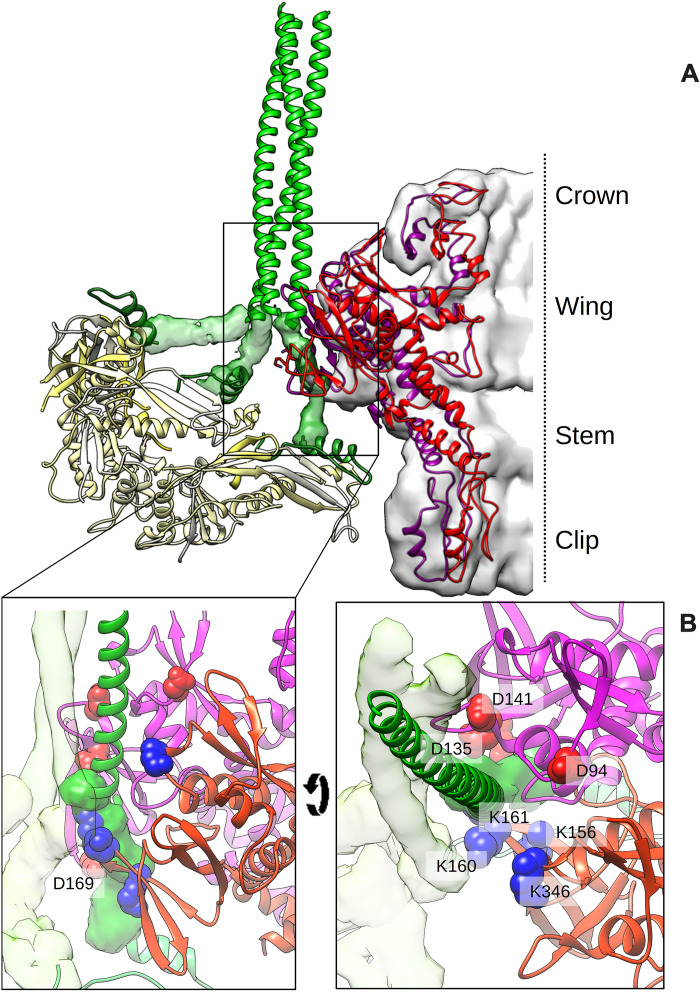
The structural model of both the mcp and the portal reveals a crucial interaction with the wing domain. (**A**) Three models of mcp are shown next to the model of the portal, showing that the portal/mcp interaction is largely between the wing domain of portal and the scaffold coiled coils. (**B**) One helix of the coiled coil (green) nestles in a groove between two portal subunits (purple and red), where it interacts with charged residues shown as van der Waal surfaces in blue and red.

The asymmetric reconstruction of the portal vertex shows that the portal and surrounding capsomer density were equally well defined (Fig. 4), indicating that the portal and the five surrounding hexons have a unique mutual orientation. Compared to the penton vertex, the hexons adjacent to the portal exhibit only slight structural changes including a small displacement (movie S2). The portal clip domains visible from the exterior (Fig. 4A) exhibit neither strict 12-fold nor 5-fold symmetry, suggesting that the 12 portal subunits adjust to their incongruent fivefold environment. Cross sections revealed stripes of density skirting the portal that do not match any reported portal structure (Fig. 4B). Visualized from the capsid interior (Fig. 4C), these stripes correspond to ten ~100-Å-long towers that surround the portal and originate from the surrounding capsomers, two towers per hexon. The towers were readily modeled as trimeric coiled coils consistent with residues 2 to 66 from mcp^N^ and accounted for all 30 scaffold domains around the portal. Besides the clip domain, the portal density exhibits strict 12-fold symmetry and the 10 towers of mcp^N^ interact at symmetrically equivalent positions on the side of the portal (Fig. 4C, right), but leaving two specific positions unoccupied. Thus, the scaffolding domains adopt a pseudo-12-fold organization, buffering the 12 to 5 symmetry mismatch at this special vertex. However, as the scaffold coiled coils originate in pairs from each hexon, they are distributed as four adjacent coiled-coil towers on one side of the portal and six on the other, bracketed by a gap between each group. Last, the helix-turn-strand motif (mcp^N^ residues 76 to 105) visualized under the regular penton vertices is also found under the portal-adjacent hexons along with additional weak density that links the ends of the spoke helices (S76) to their associated coiled-coil tower helices (S66), yielding a complete view of the entire mcp^N^ scaffolding domain adjacent to the portal (Figs. 5 and 6 and figs. S3 and S4).

Cross sections along the portal axis showed that the portal/mcp interaction is mediated by mcp^N^ (Fig. 5), including some of the surrounding S76 to S88 α helix but primarily by the flexible spacers and coiled coils that adhere to the portal’s 12-fold symmetry. Our model of the portal, despite lacking 50 N-terminal residues that are not visible in the density map, revealed several contacts with the scaffold that occur in a groove between the wing domains of two portal subunits in Fig. 6B. We observed that only one of the three coiled-coil helices interacts in this groove where it is surrounded by a polar distribution of charges (Fig. 6B): lysines (156, 160, 161, and 346) from one portal subunit and aspartates (94, 135, 141, and 169) from the other. Although the local resolution of the scaffold domain is too low in that area to assign side chains (figs. S5 and S6), we could nonetheless develop a generic coiled-coil model using CCBuilder 2.0 (*29*) to fit residues 2 to 66 into that density. The model indicates several charged residues that may participate in the scaffold/portal interaction.

## DISCUSSION

The key to understanding assembly for this class of viral capsid lies in the structure of the earliest complete procapsid, where pentons and hexons are correctly positioned on the icosahedral lattice while also incorporating the large and symmetrically inconsistent portal at one vertex. The scaffold initiates and guides this complex ballet, and its removal primes the capsid for terminase binding and the concurrent processes of DNA packaging, capsid expansion, and covalent cross-linking of mcps (*30*, *31*). Using HK97 Prohead I, where the scaffold and mcp are domains of the same protein, we resolved the structure of the scaffold and propose mechanisms by which this transient domain fulfills some of those crucial roles.

Consistent with the scaffold’s multiple functions, we resolved multiple domains that are either rigid or flexible and organized differently according to their location, i.e., under the hexon, under the penton, or contacting the portal. Notably, the full-length scaffold structure is only visible at the portal vertex where it is stabilized by the portal. Here, we observed the 12-fold portal wrapped by scaffold domains extending from the five surrounding hexons in the form of 10 towers of trimeric coiled coils that adopt a pseudo-12-fold organization, buffering the local 5 to 12 symmetry mismatch. Although unexpected, this structure explains well how the almost perfectly 12-fold portal is accommodated at the almost perfectly 5-fold vertex made of mcp^C^.

### The portal is an assembly hub

How capsids incorporate precisely one portal is a long-standing question in tailed virus assembly. The portal is often required to generate a shell of the right size and symmetry, as its absence leads to aberrant capsid structures for phages T5 and SPP1 (*18*, *32*) and tubes of mcp for phage T4 (*33*). HK97 is an exception because the mcp alone forms correctly shaped icosahedral capsids, suggesting that the HK97 portal plays a lesser role in regulating assembly, at least when the mcp is highly expressed from plasmids. Nonetheless, portal incorporation is required for virion formation and propagation, leading to the hypothesis that capsid assembly is nucleated by the portal, thus favoring shells containing a single portal. How would the portal facilitate formation of the capsid around itself? Evidence points to a fundamental role played by the scaffold. As shown for phages λ (*34*), SPP1 (*35*), P22 (*9*), T4 (*36*), and phi29 (*37*) and for the herpesvirus HSV-1 (*8*), interaction of the portal with scaffold is required for incorporation of the portal into the shell. The same dependency is implied by our model of the HK97 Prohead I portal vertex where the scaffold domains provide the major contacts between the portal and the assembled shell. We show that no less than 30 mcp^N^ domains interact directly with the portal. In comparison, the corresponding mcp^N^ domains around the regular penton vertices do not exhibit any substantial interaction with the penton subunits, suggesting that assembly of the portal vertex follows a different path to the regular penton vertices. Thus, the portal can be seen as an assembly hub around which 30 mcp subunits not only associate though the coiled-coil regions of their scaffold domains but are also recruited. The portal might select and facilitate the formation of hexons from capsomer precursors (*38*) in its vicinity that, once formed, would accrete more capsomers, accelerating the formation of a closed capsid.

### Scaffold removal acts as an assembly checkpoint

Prohead I does not expand unless treated with scaffolding-disrupting agents such as urea (*39*) or heat (*30*), suggesting that the scaffold prevents premature capsid expansion resulting in aberrant structures (*40*). A crystallographic study (*28*) found that Prohead I and Prohead II are morphologically similar except for the presence or absence of a gap at the pseudo-threefold axes. However, the 5.2-Å resolution structure of Prohead I lacked detailed subunit conformations. Our higher-resolution cryo-EM map of Prohead I shows notable differences with Prohead II, including visualization of ~25 mcp^N^ residues bound to mcp^C^, displacement of one end of the spine helix, reorganization of the P-loops, and a complete absence of N-arm density. Note that slight differences between the crystallographic Prohead I model and our density map may be due to one or several causes, including crystallization conditions and lack of a portal, compared to the cryo-EM experiment.

The remainder of the scaffolding domain is poorly structured density projecting toward the center of the Prohead, as previously reported (*11*, *19*). Our data show not only that the Prohead I P-domain is constrained (the spine helices are bent and even displaced from their positions in Prohead II) but also that the P-loops at the end of that domain adopt an arch conformation, preventing interaction with neighboring capsomers at the pseudo-threefold interfaces. At the icosahedral threefold, the P-loops interact closely with each other, but in a manner different than in Prohead II, probably because they align with the long axis of the Prohead I hexamer skew axis. The scaffold C terminus binds to both the spine helix and to the P-domain β sheet, which suggests a potential regulatory mechanism of assembly where the scaffolding domain locks the mcps of assembling capsomers into the Prohead conformation, thus constraining interactions between capsomers to specific sites that guide correct assembly and shell closure. After Prohead I is complete, digestion of the scaffold triggers a chain of events starting with the release of the scaffold constraints on the P-domains, spine helices, and N-arms, allowing local rearrangements such as movement of the P-domains toward the pseudo-threefold axes. These newly formed intercapsomer contacts provide fixed anchors for expansion (*21*). Removal of the scaffold likely affects the portal conformation even more because of the loss of portal-scaffold interactions. Examining the portal vertex in Prohead II and the subsequent stages of capsid maturation will be of particular interest to see how the 12-fold portal ring evolves at the fivefold vertex without the scaffold to buffer these two apparently incompatible environments.

Visualizing the short-lived scaffold in phage capsids has proven a challenge. However, phage HK97 is tractable in this regard because of its particular stoichiometry and the resulting steric constraints. Several clues point toward an assembly paradigm common to the double-stranded DNA–tailed phage family and herpesviruses: The key proteins involved are structurally conserved, including the canonical HK97 mcp fold, the portal and protease folds, and scaffolds that are predicted to be α-helical and to form coiled coils. Furthermore, the location of scaffold density is similar in other systems, including the scaffold binding domains of P22 (*15*) and T7 (*16*) observed under the capsomer near both the spine helix and the N-arm. Last, the scaffold domain in HK97 is somewhat independent of the mature mcp because the N-arm that links it to mcp^C^ is likely flexible as it is not visible in density maps. Hence, the HK97 scaffold domain, despite its covalent attachment to the mcp^C^, behaves as an independent protein, suggesting that the mode of scaffold expression does not affect the existence of a common assembly mechanism.

While our structural data reveal important regulatory roles for the scaffold in capsid assembly, details of how this transient assembly factor functions in time and space remain to be resolved. The triggering of scaffold proteolysis and the subsequent onset of DNA packaging is beyond the scope of this study but our work has visualized a key actor in this process that likely has relevance across the huge and ubiquitous family of double-stranded DNA–tailed phages and herpesviruses.

## MATERIALS AND METHODS

### Prohead I preparation

HK97 Prohead I particles with portals were prepared as previously outlined (*23*). Briefly, plasmid pVPB, which expresses only the wild-type HK97 portal and mcp genes *3* and *5* from a T7 promoter, was expressed in *Escherichia coli* BL21 (DE3). Cells were collected, lysed, and digested with deoxyribonuclease I, and cell debris was removed by centrifugation. Proheads were precipitated using 0.5 M salt and ~6% (w/v) polyethylene glycol, molecular weight 8000; collected by centrifugation; suspended in buffer G [20 mM tris-HCl (pH 7.5) and 100 mM NaCl]; clarified by additional centrifugation; and then pelleted in an ultracentrifuge [2 hours in Type 45 Ti rotor (Beckman Coulter, Fullerton, CA)]. Proheads were resuspended in buffer G, loaded on top of 10 to 30% (v/v) glycerol gradients in the same buffer and centrifuged in an SW32 Ti rotor (Beckman) at 32,000 rpm. Prohead dimer bands (*23*), containing only Prohead I particles with portals, were collected; concentrated by ultracentrifugation; suspended in buffer containing 100 mM KCl in 20 mM potassium phosphate (pH 7.5); and stored at 4°C.

### EM and image reconstruction

Three microliters of purified Prohead I sample was pipetted onto a freshly glow-discharged Quantifoil R2/1 grid (Quantifoil Micro Tools GmbH, Großlöbicha, Germany) and then blotted and plunge-frozen in a Vitrobot Mk 4 (Thermo Fisher Scientific, Waltham, MA, USA). Grids were mounted on a Thermo Fisher Scientific Krios 3Gi cryo–electron microscope operating at 300 kV and equipped with a Thermo Fisher Scientific Falcon 3 direct electron detecting camera. A total of 2909 movies were collected in electron-counting mode under the control of the Thermo Fisher Scientific EPU software using a total dose of 60 e/Å^2^ divided equally over 68 fractions. The magnification of ×75,000 corresponds to 1.08 Å per pixel at the sample. Particle picking was done with EMAN2 (*41*) and the reconstruction with RELION 4 (*42*), including motion correction (*43*), contrast transfer estimation (*44*), orientation determination, and particle polishing. Briefly, a density map calculated from 72,945 particles with full icosahedral symmetry imposed (I4 in RELION) was resolved to 3.6 Å. Orientations were then expanded using the relion_particle_symmetry_expansion script. Following the geometrical transformation described previously (*45*), we recalculate updated coordinates for each vertex and re-extracted them as unique particles in a smaller box, about half the size of the capsid diameter. Vertices were then three-dimensionally classified into penton (11/12th of the total) and portal (1/12th), and these datasets were subject to independent reconstructions with reduced or no symmetry imposed. The density map of the penton vertex was resolved to 3.0 Å and that of the portal vertex was resolved to 3.6- and 3.2-Å resolution for the no symmetry and 12-fold imposed map, respectively. The so-called gold-standard approach in Relion was used throughout (*42*), and we determined the local resolution using ResMap (*46*).

### Structure analysis

For mcp^C^, we based our initial mcp model on the crystallographic structure of the mcp in Prohead II (PDB ID: 3e8k) (*21*). For the part of the scaffold domain that is well resolved, the side chain density is sufficiently well resolved to anchor the amino acid sequence and to construct a de novo model using AlphaFold (*47*), UCSF Chimera (*48*), and Coot (*49*). We then refined the model into the cryo-EM density map using ChimeraX (*50*) and its Isolde implementation (*51*) as well as Coot. The models were finally analyzed using Phenix (*52*).

For the portal, an initial single subunit homology model was made on the basis of similar known portals and manually fit into the 12-fold symmetric portal map using Chimera and local real-space refinement tools in Coot, but the clip domains remained poorly defined. Comparison of the 12-fold symmetric map with the asymmetric map revealed that the clip domains appeared to each have different conformations and thus were blurred when averaged, so a new model was built, starting with a trimmed AlphaFold model of the portal. We used the initial portal model as a guide to morph a subunit of the AlphaFold model into the symmetric portal map using local rigid body and real-space refinement tools in Coot. The subunit was 12-fold replicated in Chimera and transferred to ChimeraX for manual adjustment and flexible fitting (especially of the clip domains) into the asymmetric portal map using Isolde’s tools. Phenix real-space refinement with only adenosine diphosphate was used to generate B-factors and evaluate iterations of the model. Statistics of the models are listed in table S1. The trimeric coiled-coil models for the HK97 scaffolding domain towers that interact with the portal were built using the program CCBuilder 2.0 (*29*). We used default parameters for a threefold oligomer: radius of 6.3 Å, pitch of 194 Å, and interface angle of 20°, and the input sequence was residues 2 to 66 of the mcp.
