# Supplementary material for: A symmetry mismatch unraveled: How phage HK97 scaffold flexibly accommodates a 12-fold pore at a 5-fold viral capsid vertex
Source: Sci Adv. 2023 Jun 16;9(24):eadg8868. doi: 10.1126/sciadv.adg8868 (PMC10275583; doi:10.1126/sciadv.adg8868)
Supplement: Supplementary file 1 — Figs. S1 to S7 Table S1 Legends for movies S1 and S2 [file sciadv.adg8868_sm.pdf]

Science Advances
AAAS

Supplementary Materials for

A symmetry mismatch unraveled: How phage HK97 scaffold flexibly accommodates a 12-fold pore at a 5-fold viral capsid vertex

Alexis Huet *et al.*

Corresponding author: James F. Conway, james.conway@pitt.edu

Sci. Adv. **9**, eadg8868 (2023)
DOI: 10.1126/sciadv.adg8868

The PDF file includes:

Figs S1 to S7
Table S1
Legends for movies S1 and S2

Other Supplementary Material for this manuscript includes the following:

Movies S1 and S2

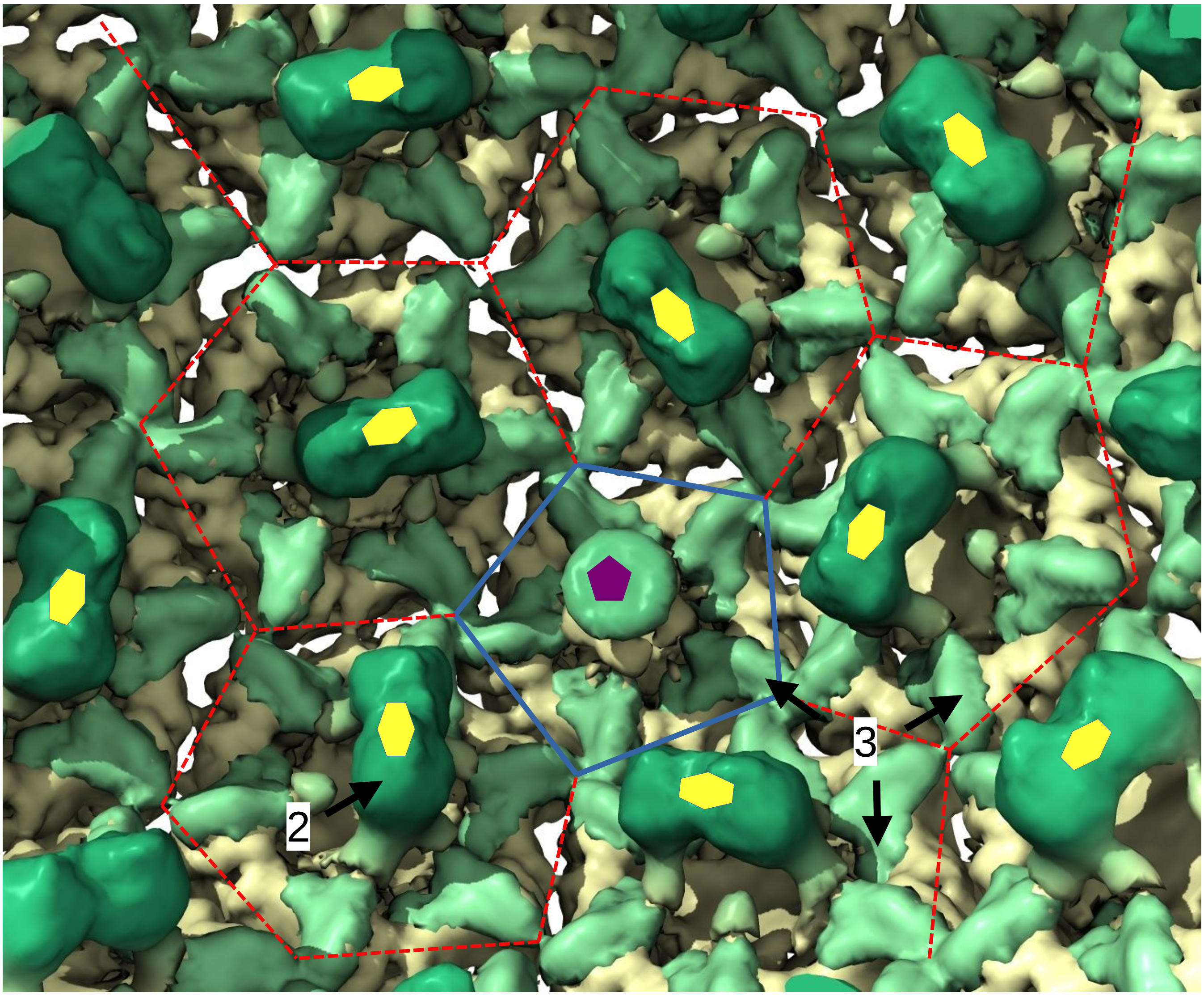

Fig S1. Interior surface view of Prohead I. A low-pass gaussian filter has been applied to the EMD-29390 map to reveal the flexible features. The scaffold projections (dark green) are clearly visible under the hexons (yellow elongated hexagon) surrounded by six spokes of density (light green) that also correspond to the scaffolding domain. Projections are also visible under the penton (magenta penton) but they exhibit a different shape. The arrows with the numbers 2 and 3 make reference to the densities seen in Fig 2A.

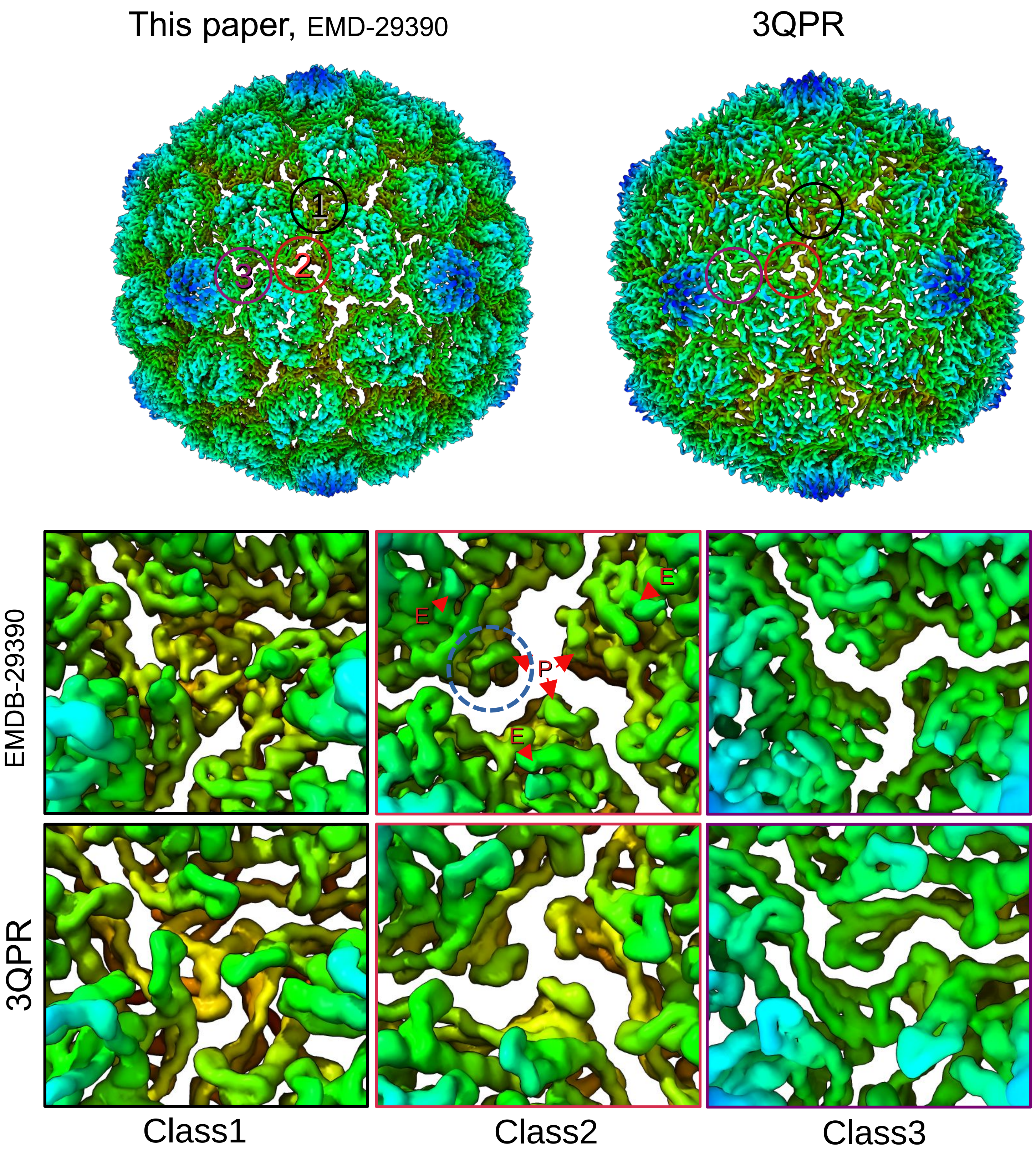

Fig S2. Comparison of the cryoEM and X-ray crystallographic Prohead I capsid structures. The Prohead I structure determined to 5.2 Å by X-ray crystallography (top row right, 3QPR) is largely similar to the cryoEM Prohead I at 3.6 Å resolution reported here (top row left, EMD-29390). However, holes are apparent at the Class2 and Class3 of our Prohead I pseudo three-fold positions (red and purple circles), but not at the Class1 icosahedral threefold (black circle) while 3QPR exhibits such a hole only at the class2 pseudo 3-fold. The holes appear to be due to the end of the P-domain (red P with arrowhead) adopting an "arch" conformation (blue dotted circle) that interacts with the E-loop (red E with arrowhead) of an adjacent subunit. For ease of comparison, our map has been gaussian filtered and the 3QPR molecular model has been rendered as a density map of 5 Å.

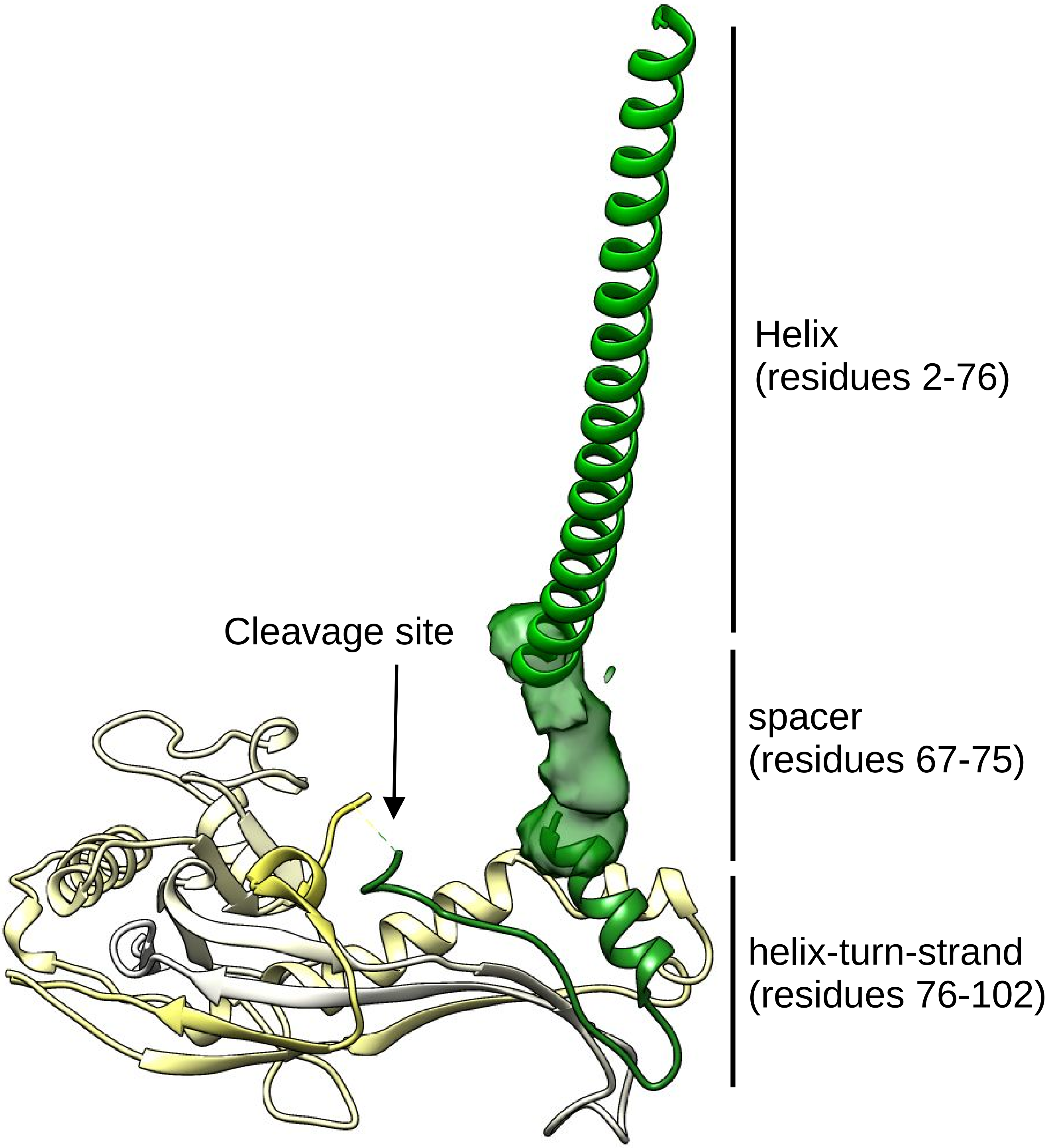

Fig S3. Modeling the portal-adjacent MCP in Prohead I. In green, the scaffold domain (mcp^N) may be divided into 3 domains: a helix that forms coiled-coils (residues 2-66) with corresponding helices from adjacent domains, a flexible spacer (residues 67-75) and helix-turn-strand domain (residues 76-102) that is rigidly bound to the capsid. In yellow, the mcp^C domain (residues 103-385) that forms the canonical HK97 fold.

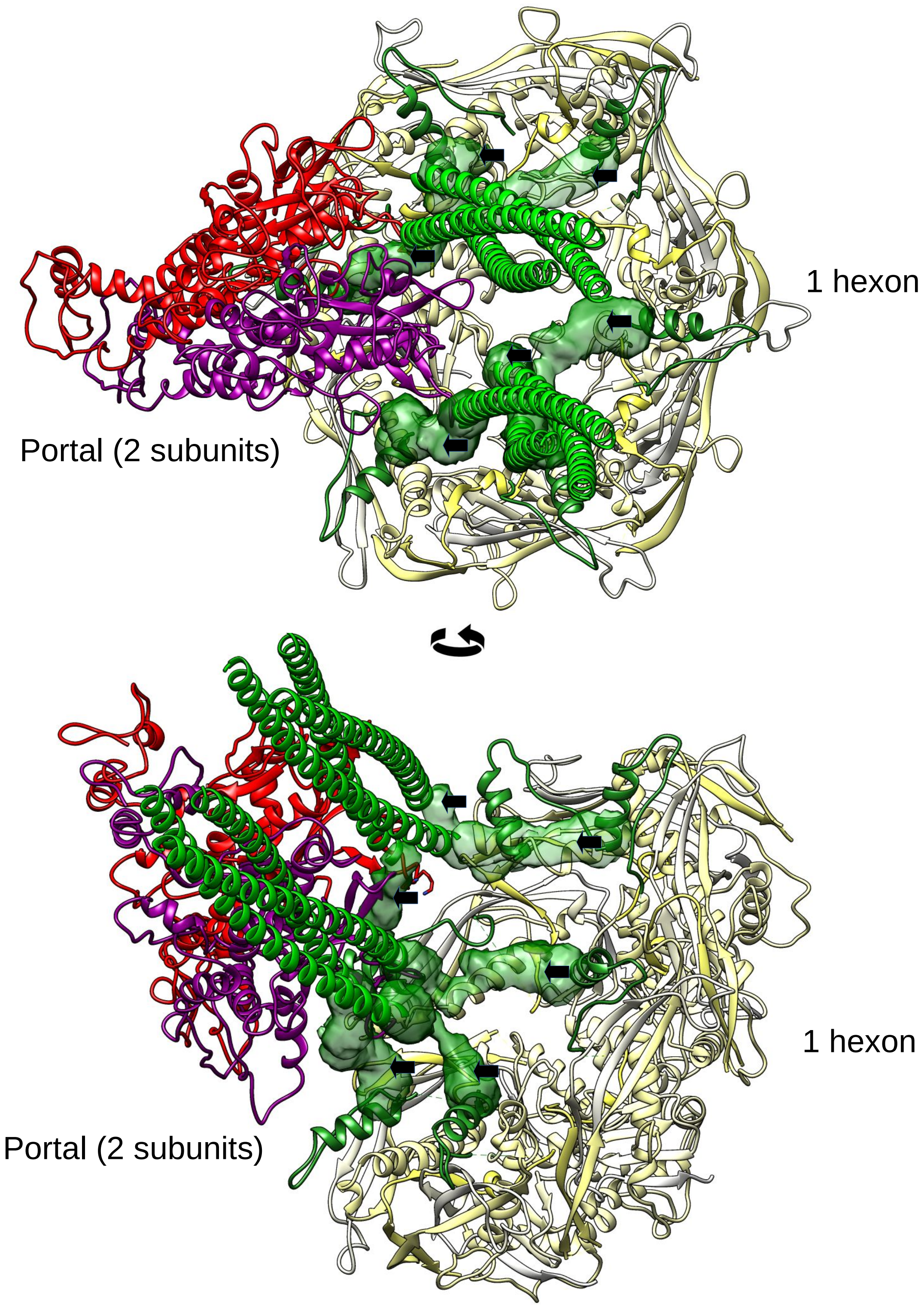

Fig S4. Assigning connectivity between the coiled-coil helices and the rigidly bound scaffold domains. Despite flexibility in the scaffold's spacer domain, the origin of each helix of the mcp^{N} coiled-coils can be assigned. Structural view of one hexon next to two subunits of portal in the prohead from the inside of the capsid, top view (top) and tilted view (bottom). The six spacers (residues ~67-75) that connect the helices of the coiled-coils (residues 1 to 66) to the rigidly bound scaffold domain (residues 76 to 102) are indicated by a black arrowhead.

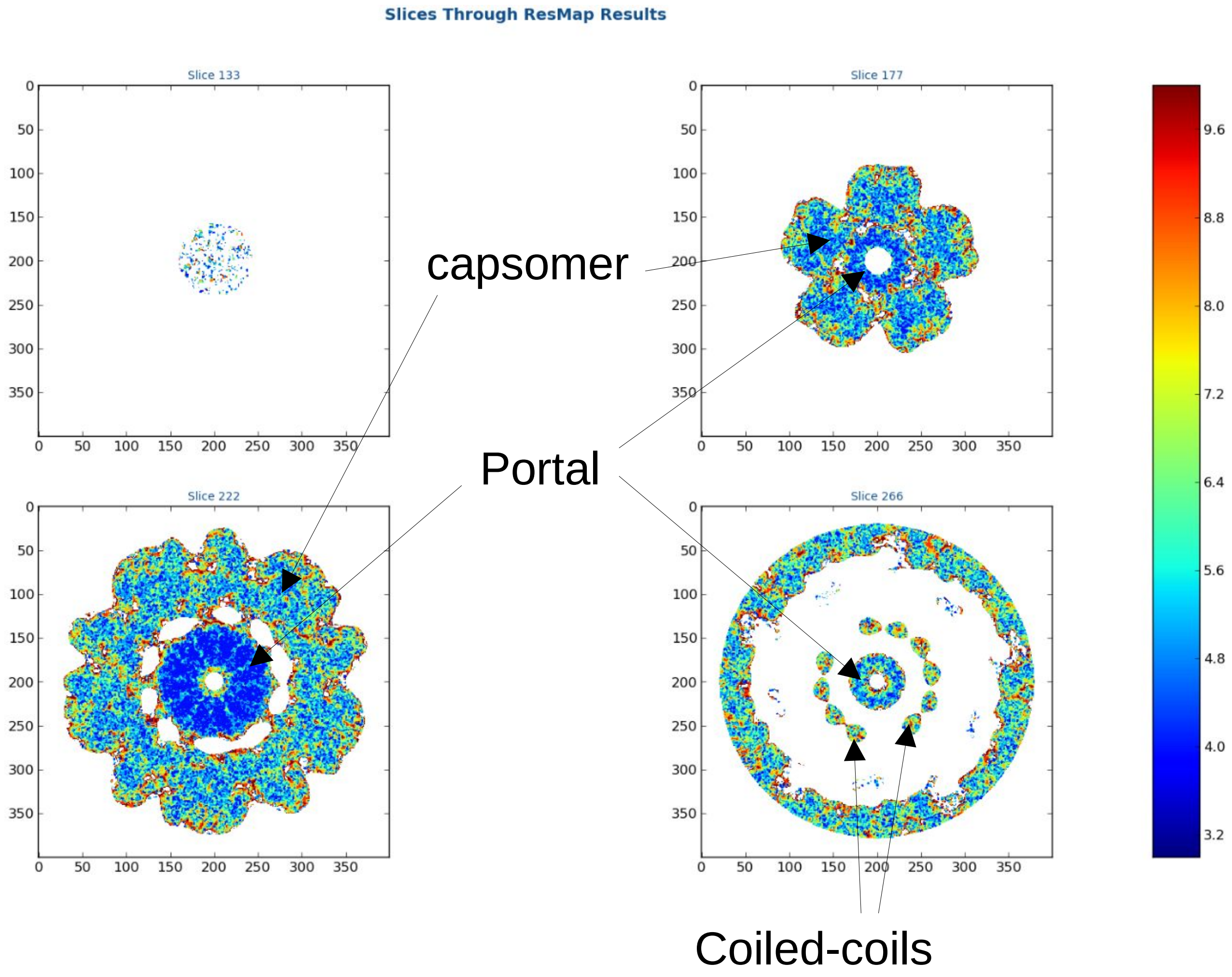

Fig S5. The portal vertex exhibits diverse levels of flexibility. The local resolution determination of the asymmetrical reconstruction of the portal vertex by ResMap (*41*) shows that the core of the portal is resoled to ~3.2Å while other structural domains such as the surrounding scaffold coiled-coils are resolved to 5-8 Å due to their flexibility.

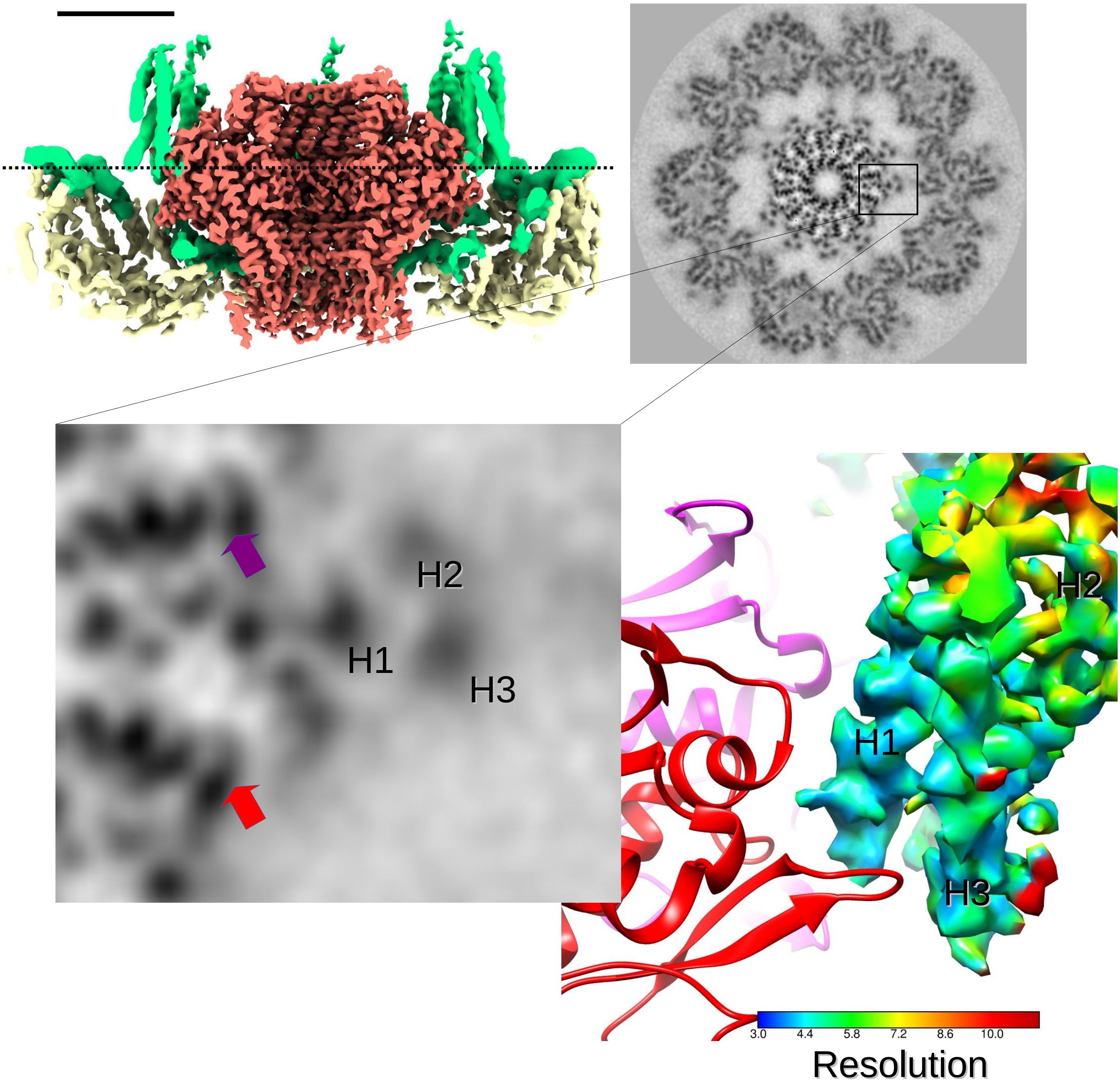

Fig S6. Flexibility of the scaffold coiled coils. The scaffold coiled-coils (residues 2-66) are not sufficiently resolved to allow reliable modeling, apparently due to flexibility. A section through the portal at the level indicated (dashed line, top left) is shown at top right, and a region enlarged at bottom left. One helix (H1) of the scaffold coiled-coil and two subunits of portal (purple and red arrows) clearly interact with each other. However, the resolution in that region is estimated to be ~5 Å, and has insufficient side chain information to establish a reliable structural model of the scaffold helices (bottom right). Scale bar is 5nm (top left)

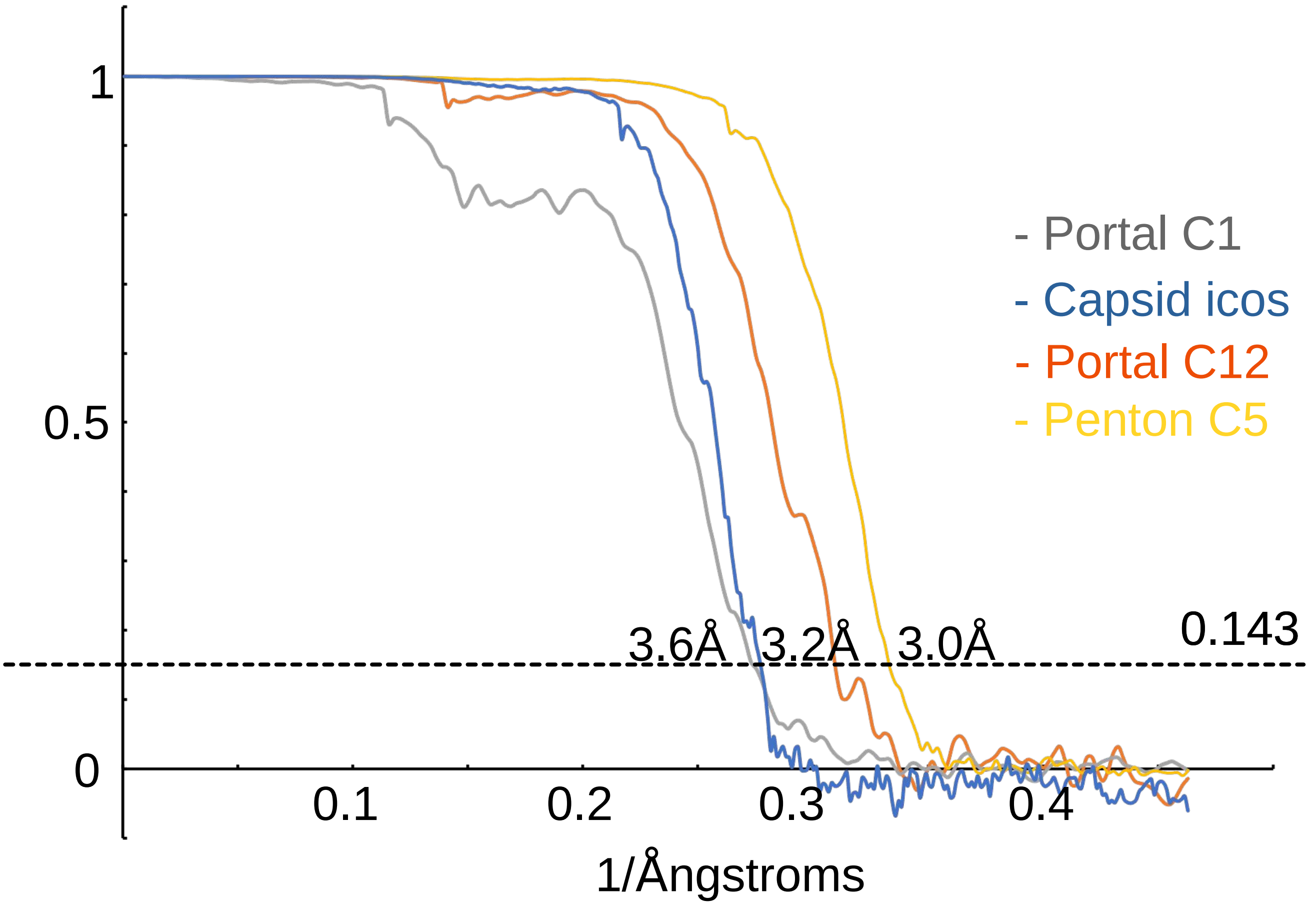

Fig S7. Resolution estimates. "Gold standard" Fourier shell correlation (FSC) curves of the icosahedral capsid, penton vertex and the portal vertex with either C12 or C1 symmetry imposed. The dashed line indicates the 0.143 threshold at which resolution is estimated.

HK97 asymmetric unit

==	
Composition (#)	
Chains	7
Atoms	28907 (Hydrogens: 14308)
Residues	Protein: 1875 Nucleotide: 0
Water	0
Ligands	0
Bonds (RMSD)	
Length (Å) (# > 4σ)	0.011 (0)
Angles (°) (# > 4σ)	1.837 (57)
MolProbity score	2.50
Clash score	5.27
Ramachandran plot (%)	
Outliers	0.76
Allowed	6.27
Favored	92.96
Rotamer outliers (%)	10.23
Cβ outliers (%)	0.35
Peptide plane (%)	
Cis proline/general	0.0/0.0
Twisted proline/general	0.0/0.1

HK97 Portal

==	
Composition (#)	
Chains	12
Atoms	32652 (Hydrogens: 0)
Residues	Protein: 4152 Nucleotide: 0
Water	0
Ligands	0
Bonds (RMSD)	
Length (Å) (# > 4σ)	0.011 (0)
Angles (°) (# > 4σ)	1.861 (133)
MolProbity score	1.65
Clash score	1.49
Ramachandran plot (%)	
Outliers	2.57
Allowed	6.64
Favored	90.79
Rotamer outliers (%)	2.01
Cβ outliers (%)	0.39
Peptide plane (%)	
Cis proline/general	0.0/0.0
Twisted proline/general	0.0/0.0

Table S1. Statistics for structural models of the asymmetric unit of HK97 (top) and the portal (bottom) calculated using Phenix.

Supplementary movie 1: From Prohead I to Prohead II. Comparison of the subunit of mcp in Prohead I (this work) to Prohead II (PDB ID 3E8K) showing that after removal of the scaffold, the subunit is subject to reshaping, notably at the spine helix and the P-loop.

Supplementary movie 2: Comparison of the regular penton and portal vertex conformations. The movie switches between the cross-sections of the portal and the penton, showing a slight displacement of the surrounding hexons between the penton and portal vertices.
